# A Molecular Subtype Model for Liver HBV-Related Hepatocellular Carcinoma Patients Based on Immune-Related Genes

**DOI:** 10.3389/fonc.2020.560229

**Published:** 2020-09-23

**Authors:** Qiyao Zhang, Xiao Yu, Qingyuan Zheng, Yuting He, Wenzhi Guo

**Affiliations:** ^1^Department of Hepatobiliary and Pancreatic Surgery, The First Affiliated Hospital of Zhengzhou University, Zhengzhou, China; ^2^Key Laboratory of Hepatobiliary and Pancreatic Surgery and Digestive Organ Transplantation of Henan Province, The First Affiliated Hospital of Zhengzhou University, Zhengzhou, China; ^3^Open and Key Laboratory of Hepatobiliary and Pancreatic Surgery and Digestive Organ Transplantation at Henan Universities, Zhengzhou, China; ^4^Henan Key Laboratory of Digestive Organ Transplantation, Zhengzhou, China

**Keywords:** HCC, immune score, prognosis, immune subtype, HBV-related

## Abstract

Hepatitis B virus (HBV)-related hepatocellular carcinoma (HCC) is one of the most common malignant tumors in the world with a very poor prognosis. Immunotyping is of great significance for predicting HCC outcomes and guiding immunotherapy. Therefore, we sought to establish a reliable prognostic model for HBV-related HCC based on immune scores. We identified immune-related modules of The Cancer Genome Atlas LIHC and GSE14520 data sets through weighted gene co-expression network analysis and evaluated HCC through a non-negative matrix factorization algorithm. Through further bioinformatics analyses, we identified causes for prognostic differences between subtypes. The results illustrate a significant difference in prognosis based on immunotypes, which may stem from metabolic disorders and increased tumor invasion associated with the high expression of genes related to stem cell characteristics. In conclusion, we identified a novel HBV-related HCC immune subtype and determined its immunological characteristics, which provides ideas for further individualized immunotherapy research.

## Introduction

Hepatocellular carcinoma (HCC) is one of the most widespread cancers globally and it has an extremely poor prognosis. Approximately 800,000 people die each year from HCC worldwide (1). Although great progress has been made in the treatment of HCC, the prognosis for HCC patients is still largely negative due to difficulties surrounding the early diagnosis and high recurrence of HCC (2–5). Hepatitis B virus (HBV) infection is one of the most significant causes of HCC in East Asia, especially in China (6, 7). Therefore, identifying reliable prognostic factors for HBV-related HCC is of great importance for the treatment of HCC.

Immune-related genes (IRGs) are a class of genes closely related to the activation and intensity of immune responses. Many studies confirm that IRGs are involved in the pathogenesis of HCC and are closely related to the survival outcome of HCC patients (8, 9). Related literature confirms the prognostic value of IRGs in HCC and establishes a molecular subtype model for HCC based on this (4, 10). However, some models focus on a single gene, and some models have overfitting problems, posing a barrier to consistent, wide-ranging clinical use. Therefore, a more complete molecular subtype model of HCC is extremely necessary.

Recent studies show that the tumor microenvironment (TME) plays an important role in tumor development and metastasis by affecting gene expression and biological behavior in tumor cells (11–16). The TME encompasses the cellular environment of the tumor, including fibroblasts, immune cells, endothelial cells, extracellular matrix, and various cytokines (17). Immune cells and stromal cells are the main components of the TME and greatly influence tumor prognosis. Currently, researchers have developed a set of algorithms called ESTIMATE that utilize gene expression data in The Cancer Genome Atlas (TCGA) database to estimate immune cell presence in malignant tumors and determine reliable molecular subtypes related to immune characteristics (14, 18). So far, this algorithm has been applied to colon cancer (19), breast cancer (20), prostate cancer (21), and glioma (22) and found to be effective. However, existing models for HCC prognosis based on immune score are still limited.

In this study, we use the ESTIMATE algorithm to analyze HBV-related HCC patient data from the TCGA and GSE14520 databases. Using weighted gene co-expression network analysis (WGCNA), immune-related modules and genes were identified, and functional enrichment analysis was performed. Subsequently, two immune subtypes were determined using a non-negative matrix factorization (NMF) algorithm based on immune-related genes related to prognosis. Our analysis highlights a significant difference in prognosis between these two subtypes. Based on key differentially expressed genes (DEGs) and functional enrichment analysis, we conclude that the prognostic differences between the two immune subtypes is due to metabolic dysfunction and increased tumor invasion associated with the high expression of genes related to stem cell characteristics of the C2 subtype. Our research provides a new model for HCC immunotyping and verifies its effectiveness, which contributes to further research on the difference of immunotherapy effects and provides a new perspective for immunotherapy.

## Materials and Methods

### Databases

All gene expression and clinical follow-up data were obtained from TCGA and Gene Expression Omnibus GSE14520 databases. Data preprocessing using screening criteria included only tumor samples containing HBV, removing samples without clinical follow-up information, removing samples without data on survival time, and removing samples without survival status. There were 145 TCGA samples and 156 GSE14520 samples after data preprocessing.

### WGCNA Analysis Based on ESTIMATE

We used the R software package ESTIMATE (1.0.13) to calculate the immune scores of 145 TCGA samples according to the published method (14). In brief, gene expression values were rank-normalized and rank-ordered, and then, empirical cumulative distribution functions of characteristic genes and other genes were calculated based on this. A statistic was calculated by integrating the differences between empirical cumulative distribution functions. We defined ssGSEA as an immune score based on characteristics related to immune cell infiltration (23, 24). The expression profiles for protein-coding genes in these 145 samples were then extracted and underwent hierarchical clustering. Five outlier samples were removed for a total of 140 remaining samples. The Pierre coefficient was used to calculate the distance between each gene and construct a weighted co-expression network using the R software package WGCNA. To ensure a scale-free network, we set the soft threshold equal to 14 and screened the co-expression module. Next, the expression matrix was converted into an adjacency matrix, and then, the adjacency matrix was converted into a topological overlap matrix (TOM). Based on TOM, an average-linkage hierarchical clustering method was used to cluster genes with 40 as the minimum number of genes for each gene network module. After determining gene modules using the dynamic shear method, we calculated the eigengenes of each module in turn and then performed cluster analysis on the modules to merge those modules closer to each other into new modules (height = 0.25, deep split = 2, and minimodule size = 40). GSE14520 data were processed using the same method. In addition, we selected genes related to immunity in the two data sets separately and used the R software package WebGestaltR (0.4.3) for KEGG and GO functional enrichment analysis.

### Identification of Molecular Typing Based on Immune Score–Related Genes

For genes related to immune scores in the two data sets, we used the coxph function in the R software package to perform single-factor cox analysis using overall survival (OS) time and survival status, respectively, and identified genes related to prognosis in both data sets. Further, we clustered HCC samples in the two data sets by NMF based on the expression levels of these genes. We selected the standard “brunet” and performed 50 iterations. The number of clusters was set as 2 to 10, and the R package NMF was used to determine the average contour width of the shared member matrix. The minimum number of members in each subclass was set to 10. The optimal number of clusters was determined according to indicators such as cophenetic, dispersion, and silhouette and was set as 2 (Supplementary Figures S1–S4).

### Functional Enrichment Analysis of DEGs in Molecular Subtypes

Differentially expressed genes between molecular subtypes were calculated separately using the limma (3.40.6) package. False discovery rate (FDR) <0.05 and log2FC > 1 were the thresholds for the TCGA data set and FDR < 0.05 and fold change (FC) >1.5 were the thresholds for the GSE14520 data set. Through the R software package WebGestaltR (0.4.3), KEGG and GO functional enrichment analysis was performed on DEGs of different molecular subtypes in the TCGA and GSE14520 data sets. Items with FDR < 0.05 were considered significantly enriched.

### Ethical Approval

This study was approved by the ethics committee at the First Affiliated Hospital of Zhengzhou University.

## Results

### WGCNA

**FIGURE 1:**
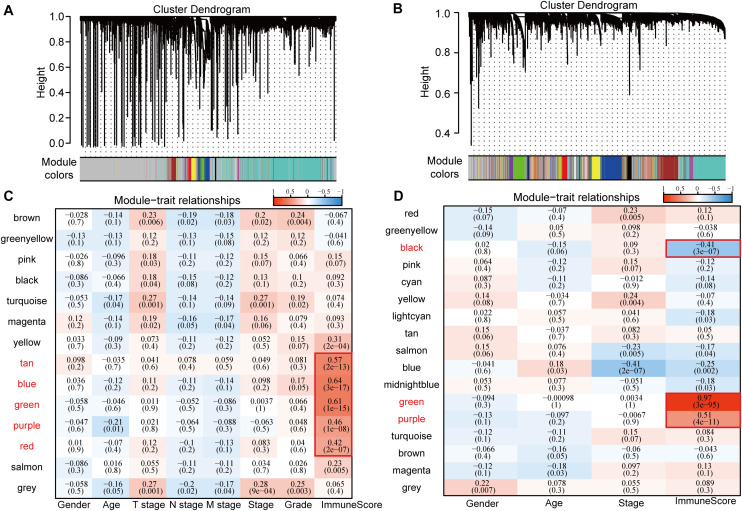
Correlation between WGCNA-based modules and sample information in HCC samples in TCGA and GSE14520 data sets. **(A)** Gene co-expression modules of the TCGA database obtained by consistent WGCNA. **(B)** Gene co-expression modules of the GSE14520 database obtained by consistent WGCNA. **(C)** Relationship between modules in the TCGA database and clinical follow-up information and immune score. **(D)** Relationship between modules in the GSE14520 database and clinical follow-up information and immune score.

Consistent WGCNA identified 14 and 17 HCC modules in the TCGA and GSE14520 data sets, respectively, (Figures 1A,B). The gray module is a collection of genes that cannot be aggregated into other modules. We further analyzed the correlation of each module with patient gender, age, TNM state, stage, grade, and immune score (Figures 1C,D). Results show that these modules in the TCGA database had no strong correlation with gender, age, TNM state, stage, or grade (cor < 0.4) although immune scores had a significantly positive correlation with the tan, blue, green, purple, and red modules (cor > 0.4, *p* < 0.00001). Gene numbers in each module included 53 in tan, 558 in blue, 261 in green, 70 in purple, and 173 in red, totaling 1115 genes. In GSE14520, in addition to a significantly negative correlation with stage in the blue module (cor < −0.4, *p* < 0.00001), the other modules had no significant correlation with gender, age, or stage (cor < 0.4) although immune scores had a significantly stronger correlation with black, green, and purple modules (cor > 0.4, *p* < 0.00001) in which the correlation between green module genes and immune score reached 0.97. Gene numbers in each module included 424 in black, 756 in green, and 320 in purple, totaling 1500 genes.

### Functional Enrichment Analysis of Genes Related to Immune Score

**FIGURE 2:**
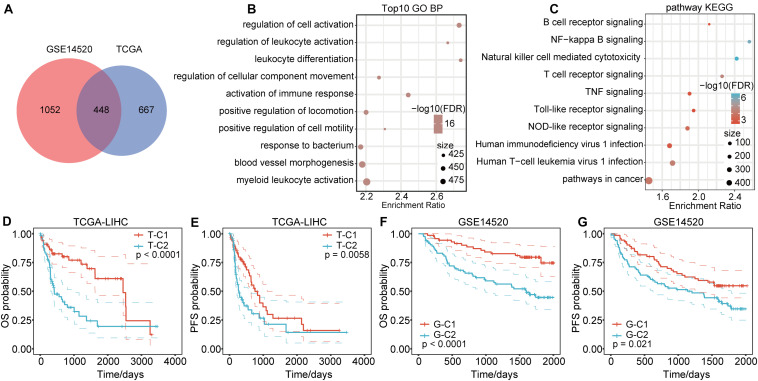
Functional enrichment pathways of immune-related genes and differential prognosis between immune subtypes. **(A)** Venn diagram of immune co-expression-related genes. **(B)** GO function annotation for BPs of immune score–related genes. **(C)** KEGG analysis of immune score-related genes. **(D,E)** The immune subtypes T-C1 and T-C2 in TCGA have significant differences in OS and PFS time. **(F,G)** The immune subtypes G-C1 and G-C2 in GSE14520 have significant differences in OS and RFS time.

Genes in the modules related to immune score in the above two databases were merged, totaling 2167 genes (Figure 2A). There were 448 genes at the intersection, accounting for 39.73% (448/1115) of genes related to immune score in TCGA and 29.87% (448/1500) of genes related to immune score in GSE14520. To identify the functions of these 2167 immune score–related genes, we performed KEGG and GO functional enrichment analysis through the R software package WebGestaltR (0.4.3). Terms with FDR < 0.05 were considered significantly enriched. For biological process (BP) immune-related functions, activation of immune response, immune response regulatory signaling pathway, and regulation of T cell activation were significantly enriched (Figure 2B). For KEGG pathway enrichment analysis, there were 69 significantly different pathways (FDR < 0.05), including natural killer cell–mediated cytotoxicity, B cell receptor signaling pathway, NF-kappa B signaling pathway, toll-like receptor pathway, T cell receptor signaling pathway, TNF signaling pathway, NOD-like receptor signaling pathway, and other immune-related pathways. Some of the annotated results are shown in Figure 2C.

### Molecular Typing Based on Immune Score–Related Genes

We extracted the expression profiles of 2167 immune score–related genes in TCGA and GSE14520 databases and used OS time and survival status to perform single factor cox analysis through the coxph function in the R software. A total of 592 genes in the TCGA data set were related to HCC prognosis (*p* < 0.05), of which 566 genes were risk factors [Hazard Ratio (HR) > 1] and 26 were protective factors (HR < 1). For the GSE14520 data set, 264 genes were related to HCC prognosis (*p* < 0.05), of which 104 genes were risk factors (HR > 1) and 160 were protective factors (HR < 1). There were 84 genes related to prognosis in both data sets.

Based on the expression levels of the 84 prognostic-related genes, the NMF algorithm was used to cluster the samples in TCGA and GSE14520. Accordingly, we divided the samples in TCGA into T-C1 and T-C2 subtypes and those in GSE14520 into G-C1 and G-C2 subtypes. Further analysis of prognostic relationships between subtypes found that by OS time and progression-free survival (PFS) time, T-C1 and T-C2 had significant differences (Figures 2D,E; log rank *p* < 0.001). Similarly, G-C1 and G-C2 had significant differences in OS time and relapse-free survival (RFS) time (Figures 2F,G; log rank *p* < 0.001). Principal component analysis (PCA) showed significant differences between different immune subtypes (Figure 4A).

### Correlation Between TNM Stage and Immune Molecular Subtype

**FIGURE 3:**
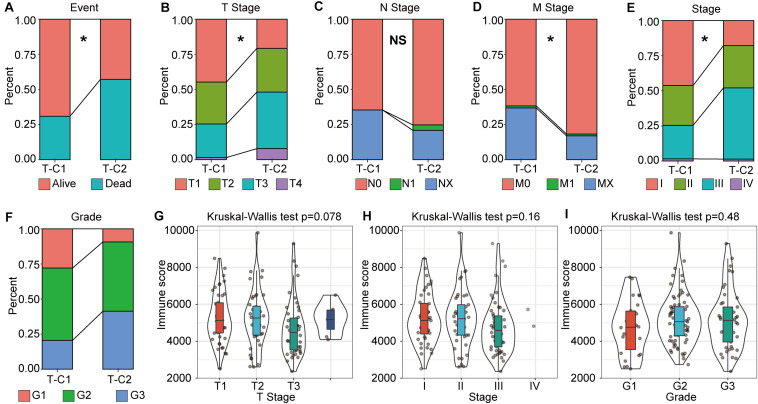
The correlation between molecular subtype, immune score, and tumor stage in TCGA-LIHC. **(A–F)** Analysis of the correlation between molecular subtypes and survival status, TNM staging, stage, and grade in TCGA-LIHC. **(G–I)** Correlation analysis between immune score and gender, tumor grade in TCGA-LIHC. *p < 0.05, “NS” means no statistical difference.

Through further analysis, we determined the correlation between immune molecular subtype, immune score and survival status, TNM stage, and grade. We found that, in TCGA-LIHC, T-C1 was associated with higher M. Stage (*p* < 0.05), and T-C2 was significantly correlated with high dead events, T. Stage, Stage, and Grade (*p* < 0.05). A higher immune score was significantly related to T. Stage (Figure 3). In the GSE14520 database, G-C2 was significantly associated with higher death events and stage (Supplementary Figure S5).

### Functional Enrichment Analysis of DEGs in Molecular Subtypes

**FIGURE 4:**
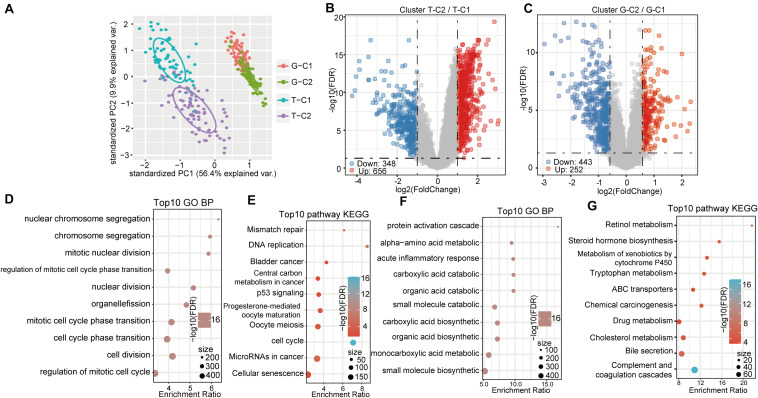
Gene expression and functional enrichment pathways between immune subtypes. **(A)** PCA diagrams of different molecular subtypes. **(B)** Volcano map of DEGs between TCGA molecular subtypes. **(C)** Volcano map of DEGs between molecular subtypes of GSE14520. **(D,E)** GO function analysis of biological processes (BPs) and KEGG analysis of genes up regulated in the T-C2 subtype in TCGA. **(F,G)** GO function analysis of BPs and KEGG analysis of genes down regulated in the T-C2 subtype in TCGA.

Differentially expressed genes between molecular subtypes were calculated using the limma (3.40.6) package. After filtering the TCGA data set (FDR < 0.05, log2FC > 1), there were a total of 1004 DEGs, including 656 up regulated and 348 down regulated genes. The difference between T-C2 and T-C1 was mainly up regulated differential expression (Figure 4B). The GSE14520 data set was filtered according to thresholds FDR < 0.05 and FC > 1.5 yielding 696 DEGs, including 253 up regulated and 443 down regulated genes. The difference between G-C1 and G-C2 was down regulated differential expression (Figure 4C).

KEGG and GO functional enrichment analysis was performed using the R software package WebGestaltR (0.4.3) on the 656 up regulated genes in the T-C2 molecular subtype from the TCGA data set, of which 230 terms were annotated to BP with significant differences (FDR < 0.05; Figure 4D). Among them, cell division, chromosome segregation, nuclear division, DNA replication, and other BPs were significantly annotated. For KEGG pathway enrichment, there were 10 significant differences (FDR < 0.05; Figure 4E), among which tumorigenesis pathways, such as mismatch repair, DNA replication, cell cycle, and p53 signaling pathway, were significantly enriched. Functional enrichment results of the DEGs down regulated in T-C2 showed that metabolic-related pathways, such as carbon metabolism, PPAR signaling pathway, tryptophan metabolism, retinol metabolism, and drug metabolism, were significantly enriched (Figures 4F,G).

**FIGURE 5:**
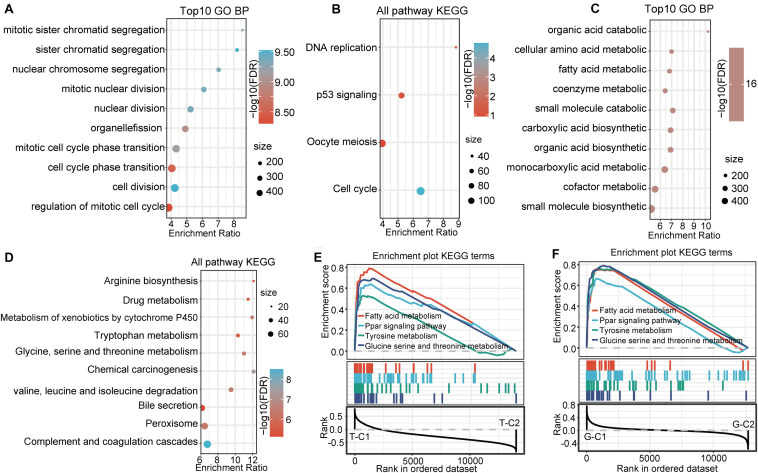
Functional enrichment pathways between immune subtypes in GSE14520. **(A,B)** GO function analysis of BPs and KEGG analysis of genes up regulated in the G-C2 subtype in GSE14520. **(C,D)** GO function analysis of BPs and KEGG analysis of genes down regulated in the G-C2 subtype in GSE14520. **(E)** Results of GSEA of immune subtypes in TCGA. **(F)** Results of GSEA of immune subtypes in GSE14520.

Functional enrichment analysis of the DEGs up regulated in G-C2 showed that 89 terms were significantly enriched in BP (Figure 5A), including cell division, chromosome segregation, nuclear division, and DNA replication. For KEGG pathway analysis (FDR < 0.05, Figure 5B), DNA replication, cell cycle, p53 signaling pathway, oocyte meiosis, and other oncogenic and development pathways were significantly enriched with the p53 signaling pathway and oocyte meiosis having significance (*p* < 0.01). Functional enrichment analysis of DEGs down regulated in G-C2 showed that tryptophan metabolism, fatty acid degradation, drug metabolism, carbon metabolism, and other metabolic-related pathways were significantly enriched (Figures 5C,D).

### Gene Set Enrichment Analysis Between Molecular Subtypes

We performed a gene set enrichment analysis (GSEA) for the subtypes in the two databases using c2.cp.kegg. v7.0 according to published methods (25). Results show that metabolic-related pathways, such as fatty acid metabolism; PPAR signaling pathway; tyrosine metabolism; glycine, serine, and threonine metabolism; and other pathways were significantly enriched in the T-C1 and G-C1 subgroups (*p* < 0.05; FDR < 0.25). Meanwhile, DNA replication, mismatch repair, homologous recombination, spliceosome, and others were found to be enriched in T-C2, and the spliceosome, cell cycle, and others were enriched in G-C2 (*p* < 0.05, FDR > 0.25; Figures 5E,F).

### Identification of Key Genes in Molecular Subtypes

**FIGURE 6:**
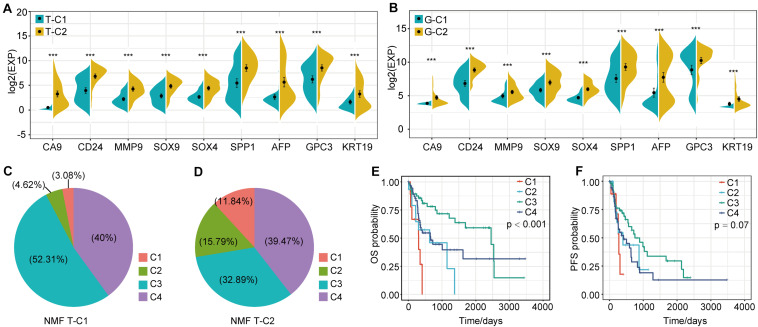
Expression differences of stem cell characteristics–related genes among immune subtypes and comparison with existing subtypes. **(A)** The expression of stem cell–related genes in molecular subtypes in the TCGA data set. **(B)** The expression of stem cell–related genes in molecular subtypes in the GSE14520 data set. **(C,D)** Proportional distribution of existing immune subtypes in T-C1 and T-C2. **(E)** KM curve of OS time of existing immune subtype in TCGA data set; **(F)** KM curve of PFS time of existing immune subtype in TCGA data set. *p < 0.05, **p < 0.01, and ***p < 0.001.

In the analysis of DEGs between molecular subtypes, we found that *SPP1*, *AFP*, *CD24*, *CA9*, and others showed significant differential expression and were highly expressed in T-C2 and G-C2. Further analysis showed that these genes were related to phenotypes associated with tumor stem cell characteristics. This indicates that molecular subtype C2 may be related to stem cell characteristics. Therefore, we screened genes related to stem cell characteristics and compared expression levels between molecular subtypes. The results showed that not only *SPP1*, *AFP*, *CD24*, and *CA9*, but also *MMP9*, *SOX4*, *SOX9*, *GPC3*, and *KRT19*, which are related to stem cell characteristics, were expressed in C2 subtypes higher than C1 subtypes (Figures 6A,B).

### Comparison Between TCGA Molecular Subtypes and Existing Immune Subtypes

Thorsson et al. performed an extensive immunogenomic analysis of more than 10,000 tumors comprising 33 diverse cancer types by utilizing data compiled by TCGA. Six immune subtypes were identified: wound healing (C1), IFN-gamma dominant (C2), inflammatory (C3), lymphocyte depleted (C4), immunologically quiet (C5), and TGF-beta dominant (C6) (26). Interestingly, we found that the proportion of C1 and C2 immune subtypes of the T-C2 subtype in TCGA increased significantly and was associated with poor prognosis, and C3 had tumor suppressive effects and a better survival rate. In our studies, C1 and C2 subtypes account for only 7.7% of the T-C1 subtype, and C1 and C2 subtypes in the T-C2 subtype account for 27.63% (Figures 6C,D). And C1 and C2 in this existing immune subtype were related to poor prognosis (Figures 6E,F). This result verifies the stability of our model.

### Comparison of Immune Scores in Molecular Subtypes

**FIGURE 7:**
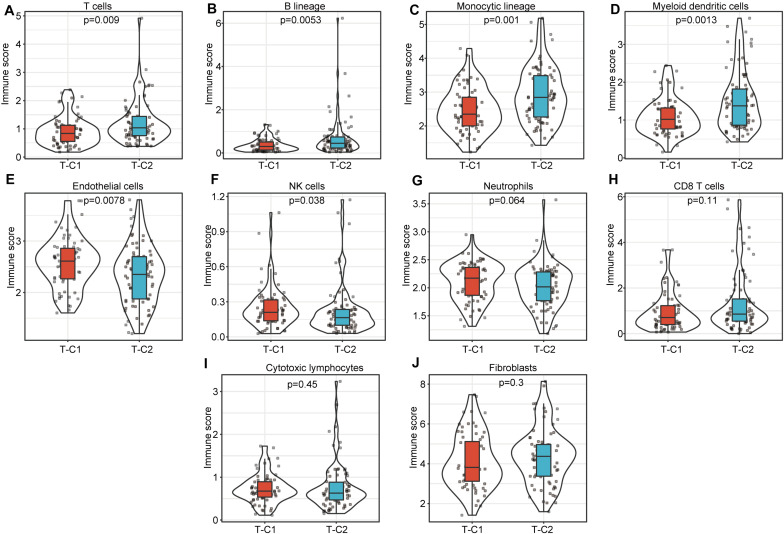
Comparison of 10 kinds of immune cells in T-C1 and T-C2. **(A–J)** Differences in immune cell scores between subtypes. T cells, B lineage, monocytic lineage, myeloid dendritic cells, endothelial cells, and NK cells had significant differences between T-C1 and T-C2 (*p* < 0.05). Neutrophils showed marginal differences (*p* = 0.064).

We compared the immune scores of the two subtype samples in the TCGA and GSE14520 data sets using the MCPcounter tool (27). The results show that T cells, B lineage, monocytic lineage, myeloid dendritic cells, endothelial cells, and NK cells had significant differences between T-C1 and T-C2 (*p* < 0.05). Neutrophils showed marginal differences (*p* = 0.064), and CD8 T cells, cytotoxic lymphocytes, and fibroblasts were not statistically different between the two molecular subtypes (*p* > 0.05; Figure 7). Among these immune cells, T-C2 had higher immune scores in T cells, B lineage, monocytic lineage, myeloid dendritic cells, and endothelial cells compared to T-C1, and NK cells and neutrophils in T-C1 had higher immune scores than T-C2. For the GSE14520 data set, only endothelial cells and fibroblasts had significant differences in immune scores with both having higher immune scores in G-C1 than in G-C2 (Supplementary Figure S6).

### Differences in Somatic Mutations Between Immune Subtypes in TCGA

**FIGURE 8:**
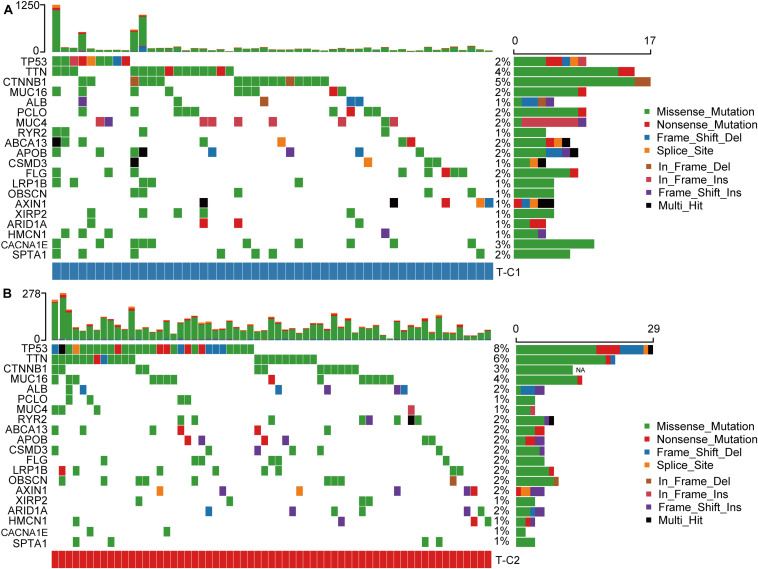
Waterfall plots of the top 20 genes with the highest frequency of somatic mutations in TCGA-LIHC in two molecular subtypes. **(A)** The mutations of the 20 genes with the highest frequency of somatic mutations in T-C1. **(B)** The mutations of the 20 genes with the highest frequency of somatic mutations in T-C2.

We drew a waterfall chart of the top 20 genes with the highest mutation frequency detected by the mutect software in the TCGA data set in two molecular subtypes, and the main type of mutation was missense mutation. The results show that the mutation rates of *TP53*, *TTN*, *CTNNB1*, *CACNA1E*, and *MUC16* are quite different among different subtypes. Among them, the mutation rate of *TP53*, *TTN*, and *MUC16* increased in T-C2, and the mutation rate of *CTNNB1* and *CACNA1E* were upregulated in T-C1 (Figure 8).

## Discussion

Studies have shown that HCC can induce an immunosuppressive TME and promote tumor progression and metastasis through multiple mechanisms (28). Immunotherapy, such as immune checkpoint inhibitors, has been shown to have effective antitumor effects. However, only a small percentage of people respond to immunotherapy (29, 30). Although there have been some studies that have conducted immunophenotyping of the prognosis of HCC, some of the research models are not stable and some have the limitation of overfitting. These studies fail to further study the immune landscape of HCC. Therefore, it is extremely necessary to explore the immunological landscape of the differential prognosis of HCC patients.

In this study, we screened hundreds of HCC samples from TCGA and GSE14520 databases. Through WGCNA analysis, we identified 14 modules and 17 modules, respectively. Further analysis showed that these modules have little correlation with patient gender, age, TNM state, stage, and grade although some of these modules had a strong correlation with immune score. This suggests that the immune score has a key role in the development and evolution of HCC. Gene enrichment analysis of genes in these immune-related modules shows that immune-related functions were significantly enriched, further confirming the above results and in accordance with previous reports (31–33).

Through single-factor analysis of immune genes, we screened 84 immune genes related to survival and then performed cluster analysis by the NMF method to divide the TCGA and GSE1450 data sets into two categories. These two subtypes had significant differences in OS and PFS (RFS) time status, and the prognosis for the T-C1 and G-C1 subtypes was obviously better than that of the T-C2 and G-C2 subtypes. Through functional enrichment analysis of DEGs between subtypes, we find that, regardless of data set, both GO and KEGG analysis show that up regulated differential genes in the C2 subtype are related to mismatch repair, DNA replication, and cell cycle functions, and down regulated differential genes are related to metabolic function. Meanwhile, we used GSEA to analyze the functions of C1 and C2 in the TCGA and GSE14520 data sets and obtained similar results to the functional enrichment of differential genes: the C2 subtype was related to mismatch repair, DNA replication, and cell cycle function, and the C1 subtype was related to metabolic function. This means that, in the C2 subtype, pathways related to tumorigenesis and development are activated, and pathways related to normal metabolism are inhibited. Many studies confirm that changes in the immune state of the TME can affect tumor metabolism and cause changes in tumor biological behavior (34–38). Based on this, we speculate that one possible reason for the poor prognosis of the C2 subtype is that normal metabolic function is inhibited, causing metabolic disorders.

Thorsson et al. analyzed the immunological characteristics of more than 10,000 samples of 33 types of cancer and showed that immunohistochemical characteristics are an important factor in predicting cancer prognosis, identifying six immune subtypes: C1 (wound healing), C2 (INF-r predominant), C3 (inflammation), C4 (lymphocyte depletion), C5 (immunologically silent), and C6 (TGF-beta predominant) of which C1, C2, and C6 are related to poor prognosis and C3 has a tumor suppressor effect and better survival rate (26). Compared with our model, we find that the proportion of C1 and C2 types (associated with poor prognosis) in T-C2 samples was significantly higher than in T-C1. This result further validates the stability of our model.

In the somatic mutation data, the mutation rate of *TP53*, *TTN*, and *MUC16* in the T-C2 subtype were up regulated of which *TP53* and *MUC16* were related to immune status. *TP53* mutations have been shown to show inflammation-related functional gains in non-small cell lung cancer and breast cancer, etc. (39, 40). *MUC16* has also been shown to be an important part of the immune genetic landscape. Its mutation is related to the increase of tumor mutation burden and may become a potential target for immune checkpoint inhibitor (ICI) therapy (41). *CTNNB1*, which has an up regulated mutation rate in T-C1, was initially shown to be associated with ICI resistance, and its evidence needs to be further studied (42).

Furthermore, we analyzed the most significant DEGs between subtypes in the two data sets and found that *SPP1*, *AFP*, *CD24*, *CA9*, and others showed the most differential expression and were highly expressed in molecular subtypes T-C2 and G-C2. Interestingly, the functions of these genes are related to tumor stem cell characteristics. As a crucial gene in tumor pathogenesis, *SPP1* is related to the stem cell characteristics of HCC and is involved in PD-L1-mediated immune escape in HCC (43). *AFP* expression mainly occurs in fetal liver cells, and although *AFP* disappears from the blood about 2 weeks after birth, its overexpression can be detected in liver cancer patients. As a marker of hypoxia, *CA9* is also a marker for poor prognosis in HCC, and recent studies show that its expression is related to stem cell phenotypes (44, 45). This indicates that molecular subtype C2 may be related to stem cell characteristics. Additionally, in the TCGA and GSE14520 data sets, the expression of genes *MMP9*, *SOX4*, *SOX9*, *GPC3*, and *KRT19* in the C2 subtypes were higher than those in the C1 subtype. All of these genes are also related to characteristics found in stem cells (44, 46, 47). Thus, we define the C2 subtype as a subtype related to stem cell characteristics. Studies show that the expression of such characteristics in tumor stem cells enhances the aggressiveness of the tumor, leading to poor prognosis. This explains the poor prognosis of the T-C2 and G-C2 subtypes, which is likely due to increased tumor invasiveness caused by increases in genes related to stem cell characteristics.

Our independent analysis of the TCGA-LIHC and GSE14520 data sets confirms that our immunophenotyping model is reliable and effective. Through bioinformatics analysis, we identified two immune subtypes with significant prognostic differences and determined the reasons. Furthermore, we demonstrated the inherent immunological characteristics of the two immune subtypes, including the differences in various immune cells and somatic mutations. This model provides a comprehensive perspective for the study of molecular subtypes of HBV-related HCC patients, and provides new ideas and basis for further research on individual differences in immunotherapy.

## Conclusion

We conducted an in-depth bioinformatic analysis on HCC samples from the TCGA and GEA14520 databases and determined new immune subtypes based on differences in immune genes. Among them, T-C2 and G-C2 subtypes have a poor prognosis, which may be due to metabolic dysfunction and increased tumor aggressiveness caused by stem cell characteristics. This is of great significance for the diagnosis of immune characteristics of patients with HBV-related HCC and the further research on personalized immunotherapy.

## Data Availability Statement

Publicly available datasets were analyzed in this study, these can be found in The Cancer Genome Atlas (https://portal.gdc.cancer.gov/); the NCBI Gene Expression Omnibus (GSE14520).

## Author Contributions

YH and WG designed the study. QiyZ and XY searched the articles and made the figures. QiyZ and QinZ wrote this manuscript. QinZ collected samples. All authors read and approved the final manuscript.

## Conflict of Interest

The authors declare that the research was conducted in the absence of any commercial or financial relationships that could be construed as a potential conflict of interest.

## Supplementary Material

FIGURES S1, S2 The parameters of 2–10 clusters in NMF algorithm clustering in TCGA-LIHC. According to the co-correlation coefficient and other parameters, the number of clusters is determined to be 2.

FIGURES S3, S4 The parameters of 2–10 clusters in NMF algorithm clustering in GSE14520. According to the co-correlation coefficient and other parameters, the number of clusters is determined to be 2.

FIGURE S5 The correlation between molecular subtype, immune score, and tumor stage in GSE14520.

FIGURE S6 Comparison of 10 types of immune cells in G-C1 and G-C2.
