# Supplementary figures and images for: A Molecular Subtype Model for Liver HBV-Related Hepatocellular Carcinoma Patients Based on Immune-Related Genes
Source: Front Oncol. 2020 Sep 23;10:560229. doi: 10.3389/fonc.2020.560229 (PMC7538624; doi:10.3389/fonc.2020.560229)

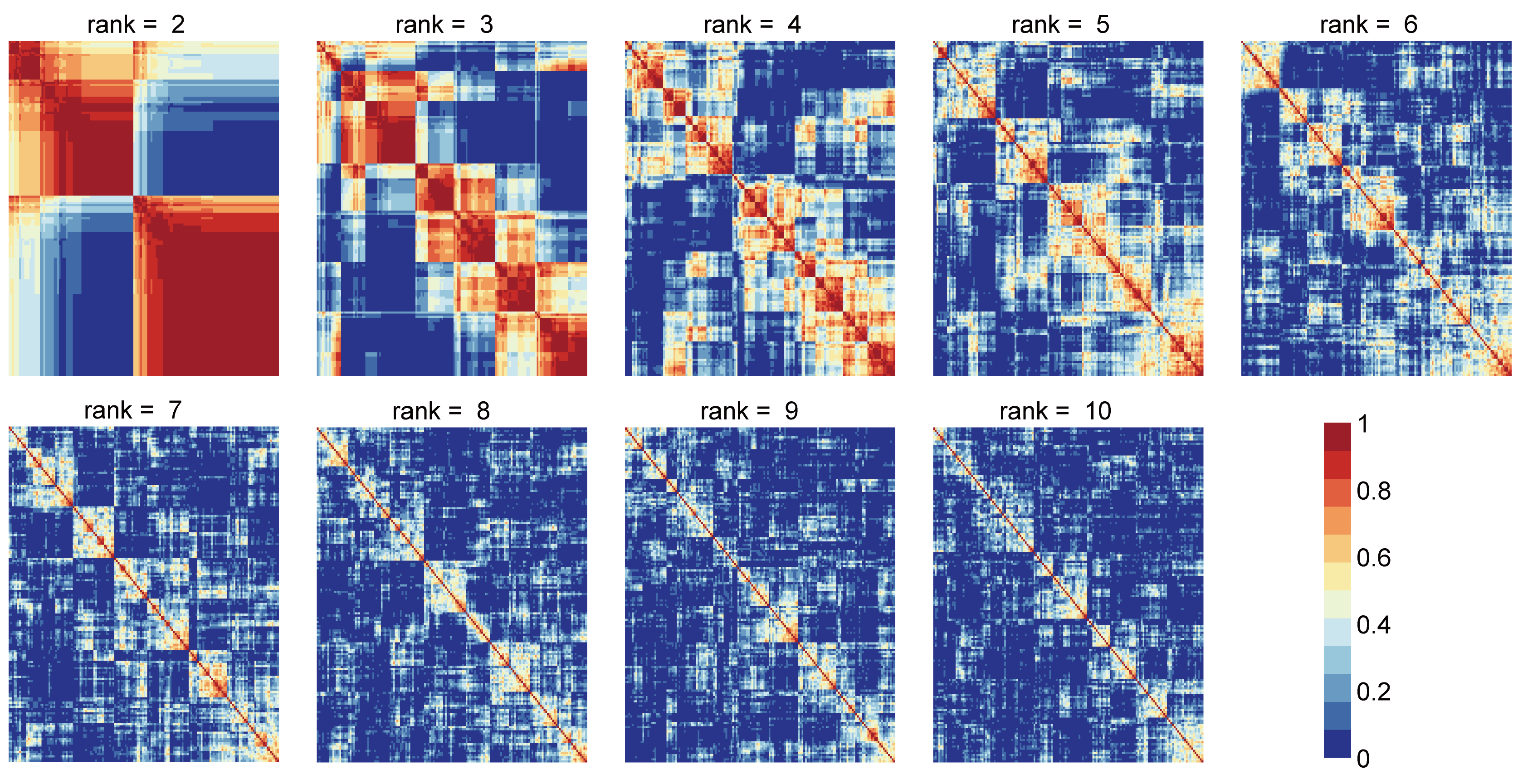

Supplement: FIGURES S1, S2 — The parameters of 2–10 clusters in NMF algorithm clustering in TCGA-LIHC. According to the co-correlation coefficient and other parameters, the number of clusters is determined to be 2. [file Image_1.TIF]

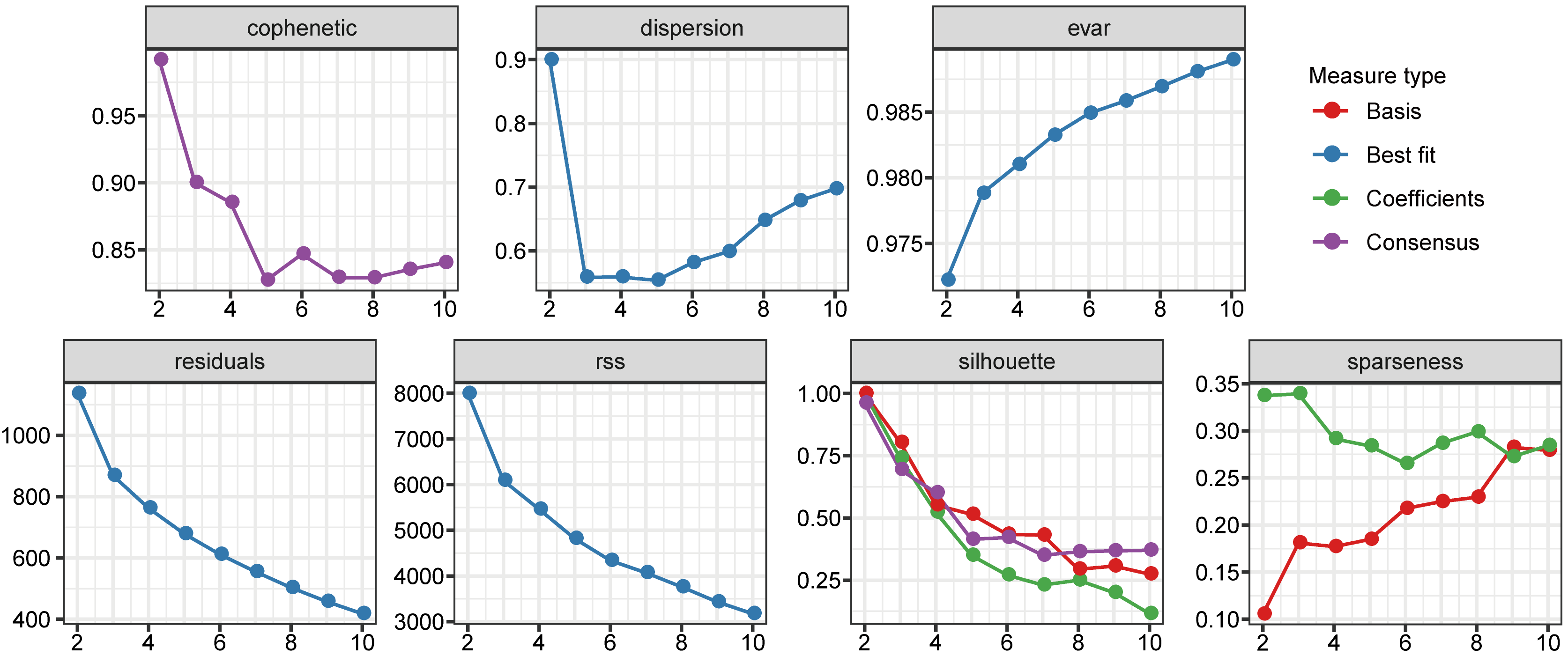

Supplement: Supplementary file 2 [file Image_2.TIF]

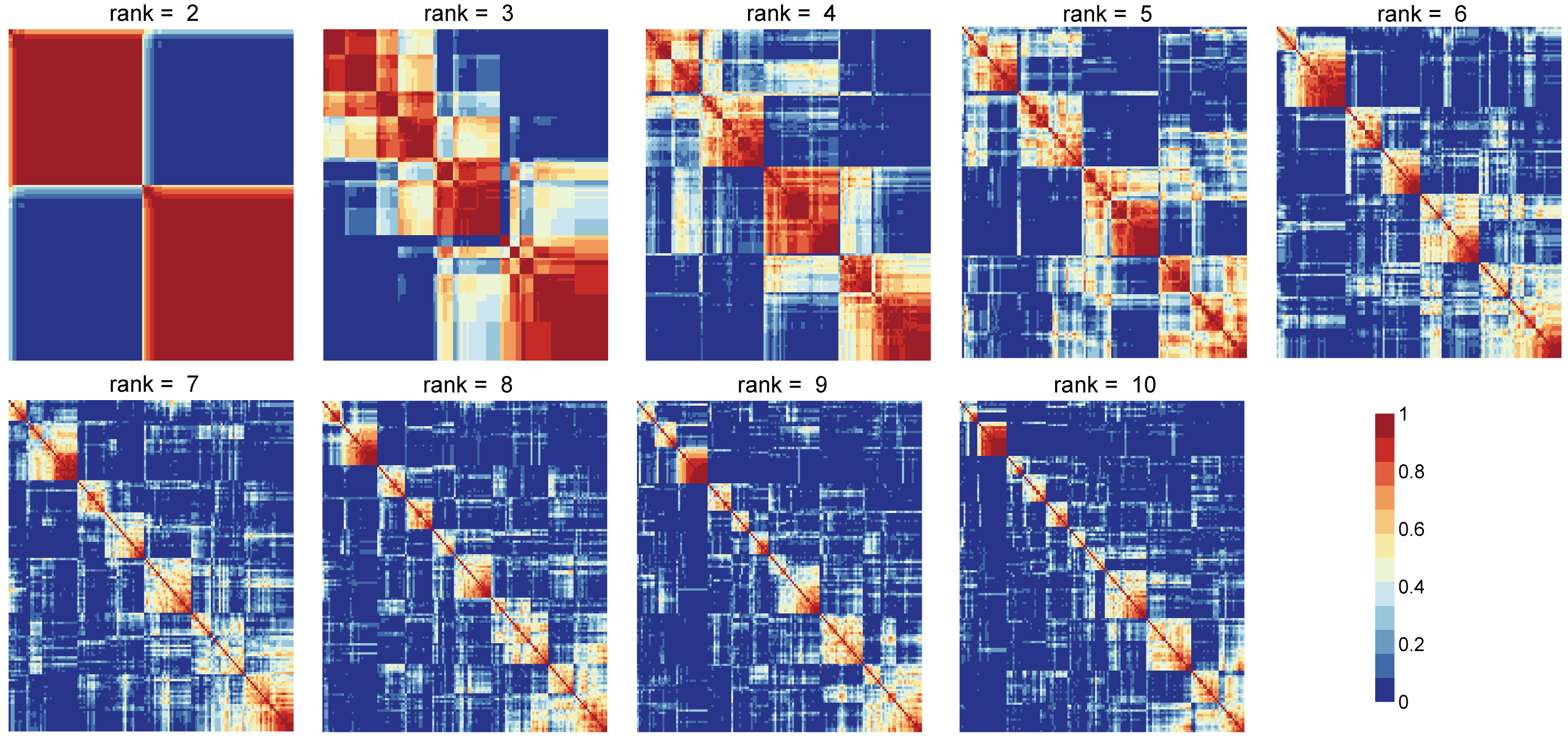

Supplement: FIGURES S3, S4 — The parameters of 2–10 clusters in NMF algorithm clustering in GSE14520. According to the co-correlation coefficient and other parameters, the number of clusters is determined to be 2. [file Image_3.TIF]

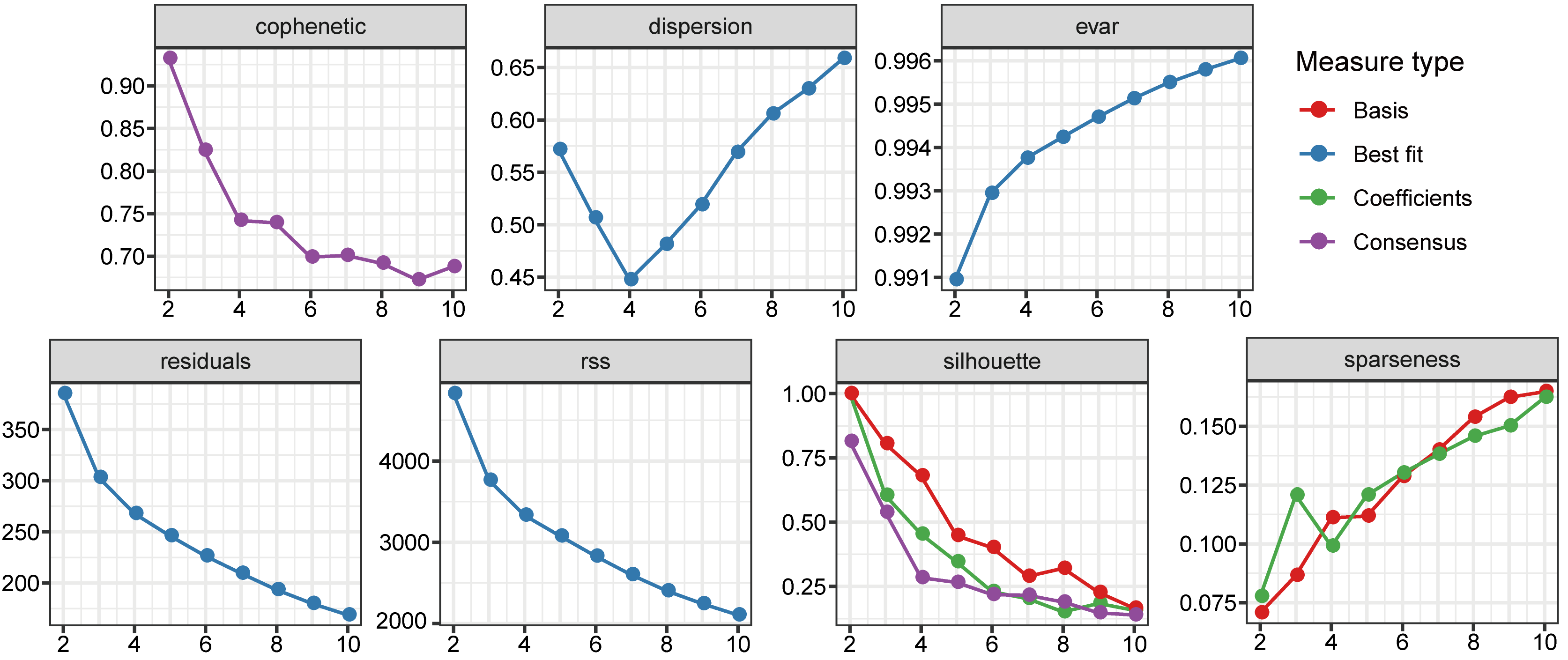

Supplement: Supplementary file 4 [file Image_4.TIF]

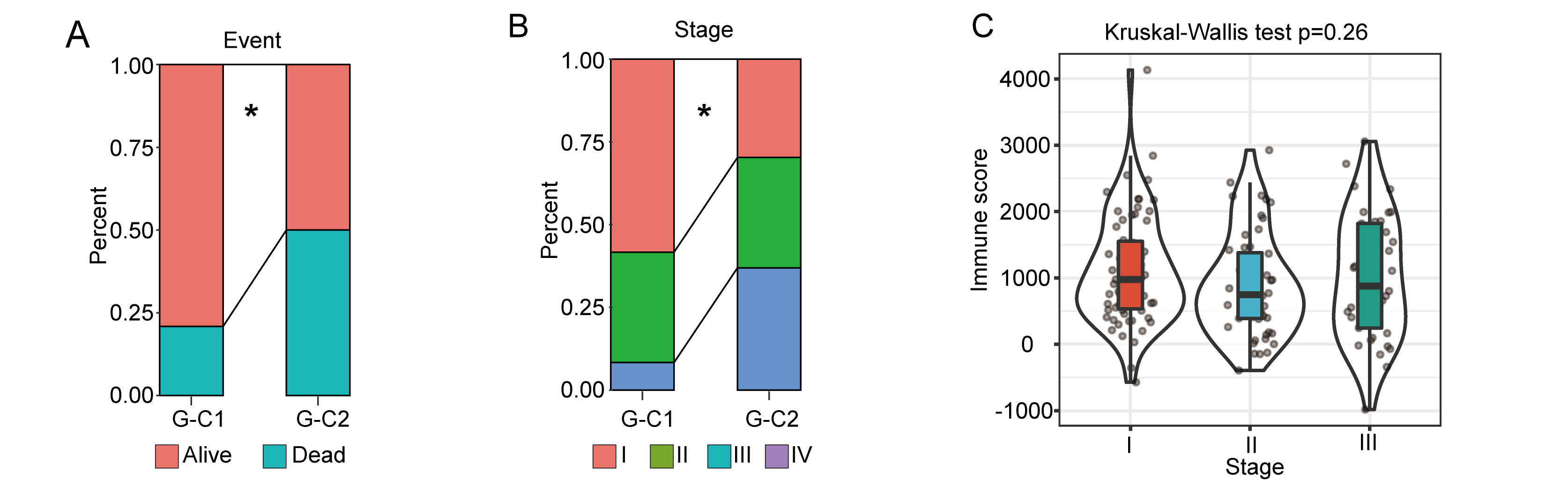

Supplement: FIGURE S5 — The correlation between molecular subtype, immune score, and tumor stage in GSE14520. [file Image_5.TIF]

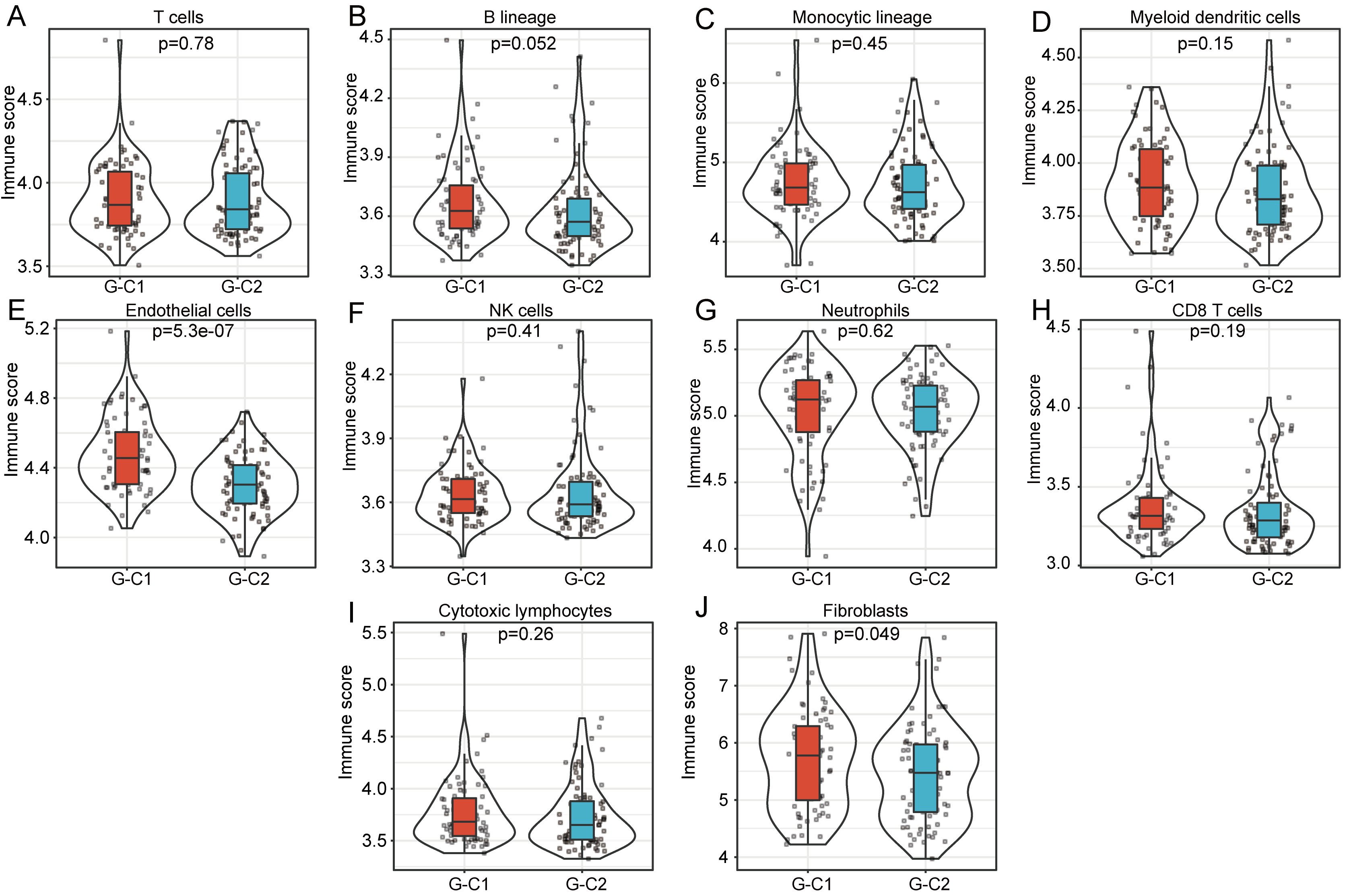

Supplement: FIGURE S6 — Comparison of 10 types of immune cells in G-C1 and G-C2. [file Image_6.TIF]
